# HPLC-DAD finger printing, antioxidant, cholinesterase, and α-glucosidase inhibitory potentials of a novel plant *Olax nana*

**DOI:** 10.1186/s12906-017-2057-9

**Published:** 2018-01-03

**Authors:** Muhammad Ovais, Muhammad Ayaz, Ali Talha Khalil, Sayed Afzal Shah, Muhammad Saeed Jan, Abida Raza, Muhammad Shahid, Zabta Khan Shinwari

**Affiliations:** 1 Department of Biotechnology, Quaid-i-Azam University, Islamabad, 44000 Pakistan; 2 Key Laboratory for Biomedical Effects of Nanomaterials and Nanosafety, CAS Center for Excellence in Nanoscience; National Center for Nanoscience and Technology, Beijing, 100190 China; 3 Department of Pharmacy, University of Malakand, Khyber Pakhtunkhwa (KPK), Chakdara, 18000 Pakistan; 4 Department of Eastern Medicine and Surgery, Qarshi University, Lahore, Pakistan; 5 Department of Plant Sciences, Quaid-i-Azam University, Islamabad, 44000 Pakistan; 6 National Institute for Lasers and Optronics (NILOP), Pakistan Atomic Energy Commission, Islamabad, 44000 Pakistan; 7 Department of Pharmacy, Sarhad University of Science and Information Technology, Peshawar, Pakistan; 8 Pakistan Academy of Sciences, Islamabad, 44000 Pakistan

**Keywords:** *Olax nana*, Phytochemical analysis, HPLC-dad, Acetylcholinesterase (AChE), Butyrylcholinesterase (BChE), DPPH, H_2_O_2_, ABTS, α-glucosidase inhibitory assays

## Abstract

**Background:**

The medicinal importance of a novel plant *Olax nana* Wall. ex Benth. (family: *Olacaceae*) was revealed for the first time via HPLC-DAD finger printing, qualitative phytochemical analysis, antioxidant, cholinesterase, and α-glucosidase inhibitory assays.

**Methods:**

The crude methanolic extract of *O. nana* (ON-Cr) was subjected to qualitative phytochemical analysis and HPLC-DAD finger printing. The antioxidant potential of ON-Cr was assessed via 1,1-diphenyl,2-picrylhydrazyl (DPPH), 2,2-azinobis[3-ethylbenzthiazoline]-6-sulfonic acid (ABTS) and hydrogen peroxide (H_2_O_2_) free radical scavenging assays. Furthermore, acetylcholinesterase (AChE) & butyrylcholinesterase (BChE) inhibitory activities were performed using Ellman’s assay, while α- glucosidase inhibitory assay was carried out using a standard protocol.

**Results:**

The qualitative phytochemical analysis of ON-Cr revealed the presence of secondary metabolites like alkaloids, flavonoids, tannins, sterols, saponins and terpenoids. The HPLC-DAD finger printing revealed the presence of 40 potential compounds in ON-Cr. Considerable anti-radical activities was revealed by ON-Cr in the DPPH, ABTS and H_2_O_2_ free radical scavenging assays with IC_50_ values of 71.46, 72.55 and 92.33 μg/mL, respectively. Furthermore, ON-Cr showed potent AChE and BChE inhibitory potentials as indicated by their IC_50_ values of 33.2 and 55.36 μg/mL, respectively. In the α-glucosidase inhibition assay, ON-Cr exhibited moderate inhibitory propensity with an IC_50_ value of 639.89 μg/mL.

**Conclusions:**

This study investigated *Olax nana* for the first time for detailed qualitative phytochemical tests, HPLC-DAD finger printing analysis, antioxidant, anticholinesterase and α-glucosidase inhibition assays. The antioxidant and cholinesterase inhibitory results were considerable and can provide scientific basis for further studies on the neuroprotective and anti-Alzheimer’s potentials of this plant. ON-Cr may further be subjected to fractionation and polarity guided fractionation to narrow down the search for isolation of bioactive compounds.

## Background

Humans have used medicinal plants as a remedy against various diseases since time immemorial [1]. The presence of various bioactive components in different parts of plants make them an important resource for the treatment of various diseases. Furthermore, the side effects associated with some of the synthetic medications and antibiotic resistance demands intermittent research for alternative solutions [2, 3]. In addition, to their direct therapeutic use, medicinal plants have been explored to fabricate various nanoscaled materials for potential biomedical applications [4–9]. Natural products based alternative therapies are still practiced in many countries and globally approximately 80% of population trust on herbal medicine as a primary source of therapeutic remedies [10]. In the last decade, a revival has been observed in the use of medicinal plants and herbal medicines. It is forecasted that the global market for herbal medicine will expand over time due to the preference of consumers for natural medicines [11]. Moreover, by the year 2017, the worldwide herbal supplements and remedies market is forecasted to reach $107 billion [12].

The genus *Olax* belongs to family *Olacaceae* [13], which contains several medicinally important plants [14]. Among the *Olacaceae* species, *Olax subscorpioidea*, is reported to have applications in the management of analgesic, cancer, yellow fever, inflammatory diseases, mental illness, Alzheimer’s disease, parasitic, microbial infections and hepatological disorders [14–23]. *Olax scandens* revealed the presence of highly useful phytochemicals like sitosterol, octacosanol, oleanolic acid, aleanolic acid and β-sitosterol [24]*.* Several studies confirmed the pharmacological importance of *O. scandens* in bacterial infections, cancer, headache and psoriasis [25–27]. Moreover, the aqueous extract of *Olax zeylanica* was found effective against skin diseases and exhibits photoprotective activity, whereas, *Olax dissitiflora* have mosquito repellent properties [28, 29]. The phytochemical analysis of *Olax mannii* Oliv revealed the presence of three new flavonoid triglycosides including kaempferol 3-O-[β-D-glucopyranosyl-(1–2)-α-L-arabinofuranoside]-7-O-α-L-rhamnopyranoside, kaempferol 3-O-[β-D-arabinopyranosyl-(1–4)-α-L-rhamnopyranoside]-7-O-α-L-rhamnopyranoside, kaempferol 3-O-[α-D-apiofuranosyl-(1–2)-α-L-arabinofuranoside]-7-O-α-L-rhamnopyranoside, kaempferol 3-O- α-L-rhamnopyranoside and fourteen already known flavonoids glycosides which are potential anti-cancer and anti-inflammatory agents [30].

Free radicals are implicated in the progression of several disorders like, cancer, ischemic heart diseases, neurodegenerative diseases, diabetics, reperfusion injuries, arthritis and atherosclerosis [31]. Free radicals from various sources like toxins, environmental pollutants and deep fried foods cause abnormal genes expression and proteins synthesis which initiate degenerative reactions in the body [32]. In the living systems, generation of free radicals occur via oxidation process, which are nullified to non-radical forms in the human body by natural antioxidants. Hence, apart from our own immune system which acts against these free radicals, natural antioxidants are of prime importance to counter them [33]. Synthetic antioxidants like tertiary butyl hydro quinone, propyl gallate, butylated hydroxytoluene and butylated hydroxyanisole are associated with adverse effects to human health. Hence, natural antioxidants form plants are found to be best alternative for synthetic antioxidants [33]. Many published studies validate the potential antioxidant potential of medicinal plants crude extracts and of isolated pure compounds [34, 35]. Flavonoids and phenolics are considered as potent antioxidants due to the presence of hydroxyl groups and conjugated ring structures, which via hydrogenation or complexation scavenge free radicals [36].

Alzheimer’s disease (AD) is a common neurodegenerative disorder characterized by behavioral turbulence, cognitive dysfunction and imperfection in the routine activities. Its prevalence is high among individuals of age above sixty years [37]. Currently only five drugs have been approved for clinical use, among which four are cholinesterase inhibitors [38]. The cholinesterase’s including acetyl cholinesterase (AChE) and butyrycholinesterase (BChE) catalytically metabolize acetylcholine (ACh) in the synaptic cleft [39]. Acetylcholine is an important neurotransmitter involved in the transmission of impulses across the synapse and is vital in the acquisition and storage of memory. The level of ACh has been found to be depleted in AD [40]. Among the options is the use of inhibitors of AChE and BChE which will restore the activity of ACh at the synapse. Among the clinically approved cholinesterase inhibitors, two are from natural products including galantamine and rivastigmine. Consequently, natural products are under consideration for the development of more useful cholinesterase inhibitors [41].

Diabetes is a major health problem which is associated with high blood glucose level in the body. The α-glucosidase enzyme plays a key role in the control of glucose level inside the body as they are related to postprandial glucose excursion in a person suffering from diabetes [42, 43]. α-Glucosidase breakdown carbohydrates into glucose and therefore increases the glucose level inside the body. Henceforth, α-glucosidase inhibition (AGI’s) is considered as a popular strategy for controlling post prandial glucose level. Many plants have been researched for natural AGI’s like *Punica granatum, Pine bark* and *Andrographis paniculata* [44].

To the best of our information there is no single report on the phytochemical investigation and pharmacogonostic features (anticholinesterase, antioxidant and α-glucosidase inhibitory potential) of *O. nana*. Therefore, the present research was undertaken to investigate and scientifically validate the use of *O. nana* aqueous methanolic extract against ailments such as diabetes and neurogenerative diseases.

## Methods

### Plant collection and extraction

**Fig. 1:**
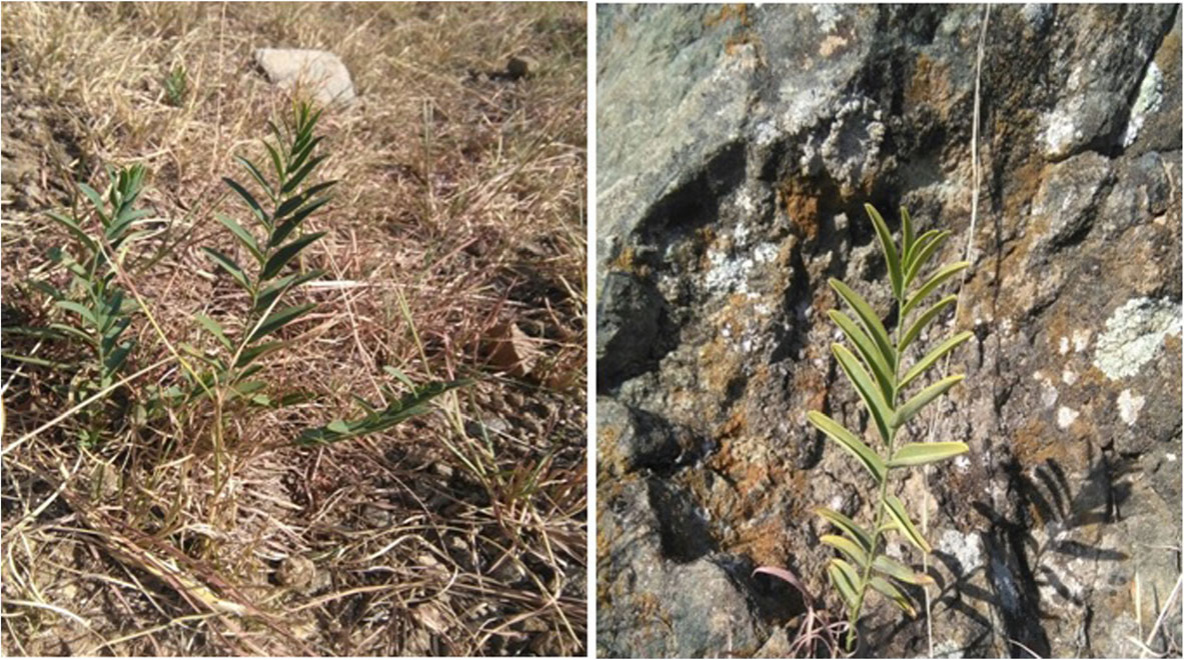
Snapshot of *Olax nana* plant at collection point (Location: Swat district of Khyber Pakhtunkhwa, Pakistan)

*O. nana* whole plant was collected from Swat district of Khyber Pakhtunkhwa, Pakistan in June 2016 (Fig. 1). The plant was authenticated by botanical taxonomist Syed Afzal Shah (PhD Candidate) and Dr. Mushtaq (plant taxonomist) at the Department of Plant Sciences, Quaid-i-Azam University, Islamabad, Pakistan. A sample was deposited at MOSEAL Laboratory, Department of Biotechnology, Quaid-i-Azam University with a voucher number MOSEAL-344. The plant was gently rinsed with running distilled water followed by shade drying for 10 days. Subsequently, the dried leaves were grinded via cutter mill to obtain a fine powdered material that weigh ~1 kg. In a 1 L of 70% aqueous methanol, 300 g of the powdered plant was soaked for 5 days in a shaking incubator (37 °C), with frequent sonication (60 Hz for 5 mints every day) using a sonicator (Elmasonic E 60 H - Cousins UK). The extraction step was repeated thrice, with the addition of extract to the original one and filtered via muslin cloth, ultimately followed by final filtration through Whatman® qualitative filter paper, Grade 1. Furthermore, in a rotary evaporator (EYELA N-1300S-W 115 V, Tokyo Rikakikai Co., LTD), the crude methanolic extract (ON-Cr) was concentrated, ultimately resulted in a ~20 g mass of dark brownish semisolid [45].

### Chemical and drugs

For antioxidant assays, 1,1-diphenyl, 2-picrylhydrazyl (DPPH) and 2, 2-azinobis [3-ethylbenzthiazoline]-6-sulfonic acid (ABTS) (CAS 1898–66-4 and CAS 30931–67-0 Sigma Aldrich, USA,K_2_S_2_O_4_ (Riedel-de Haen Germany) were purchased from authorized dealers in Pakistan. For anticholinesterase and α-glucosidase studies, AChE from *Electric eel* (type-VI-S, CAS 9000–81-1), BChE from equine serum lyophilized (CAS 9001–08-5) and α-glucosidase from *Saccharomyces cerevisiae* CAS 9001–42-7 were purchased from Sigma-Aldrich GmbH USA. The substrates including acetylthiocholine iodide (CAS1866–15-5), butyrylthiocholine iodide (CAS 2494–56-6), *p*-nitrophenyl-α-D-glucopyranoside glucopyranoside (CAS 2492–87-7), were purchased from Sigma-Aldrich UK and Sigma-Aldrich, Switzerland, respectively. Similarly, the indicator substance, 5,5-dithio-bis-nitrobenzoic acid (DTNB) CAS 69–78-3 was purchased from Sigma-Aldrich Germany. The standard drugs, galantamine hydrobromide, *Lycoris* Sp. (CAS 1953–04-4), acarbose alfa aesar (CAS 56180–94-0) and ascorbic acid were purchased from Sigma-Aldrich France. The buffer system including (K_2_HPO_4_), (KH2PO_4_), KOH and solvents used were of extra pure quality.

### Phytochemical analysis

#### Preliminary phytochemical tests

For the identification of various secondary metabolites including alkaloids, flavonoids, glycosides, saponins, sterols, tannins, terpenoids and anthraquinones, qualitative phytochemical tests were performed following standard protocols as reported previously [46].

#### Determination of alkaloids (Dragendorff’s method)

The crude methanolic extract (0.2 g) was taken in a conical flask and sulphuric acid (2%) was added. The conical flask was placed on a hot water bath for 2 min and then cooled. The sample was then filtered and treated with Dragendorff’s reagent in a test tube. The orange red color precipitate in the test tube showed the presence of alkaloids.

#### Test for flavonoids

In a conical flask, dried powdered material was boiled with 10 mL of distilled water in a hot water bath for about 5 min and then filtered. After cooling the mixture, few drops of sodium hydroxide solution (20%) was added to 1 mL of the cooled filtrate, which changed the color to yellow. Further addition of 2% HCl changed the yellow color of mixture to colorless.

#### Test for tannins

To 2 g of sample, 20 mL of distilled water was added and boiled for about 5 min in a hot water bath. The boiled solution was filtered immediately and then cooled at room temperature. A 1 mL of filtrate was taken and added to 5 mL of distilled water. Then 10% of ferric chloride (2–3 drops) was added. The appearance of bluish-black precipitate or brownish-green color showed the presence of tannins.

#### Test for anthraquinones

A 2 g powdered plant sample was taken and macerated with ether. The solution was shaken and checked, the appearance of red, violet or pink color in the aqueous layer indicated the presence of anthraquinone.

#### Test for Saponins

The powdered plant material (0.2 g) along with 10 mL of distilled water was taken in a conical flask and boiled in a water bath for about 10 min. The hot mixture was then filtered and allowed to cool. A 1 mL of filtrate along with 2 mL of distilled water was taken in a test tube and vigorously shaken for 2 min. The formation of froth indicates the presence of saponins in the filtrate. Olive oil was then added drop wise to the froth and after shaking the mixture, a formation of emulsion was observed.

#### Test for glycosides

To a test tube containing a mixture of concentrated sulphuric acid (1 mL), aqueous plant extract (5 mL) and glacial acetic acid (2 mL), a drop of ferric chloride was added. The concentrated sulphuric acid remained beneath the mixture. The appearance of brown ring indicated the presence of cardiac glycoside.

#### Test for Terpenoids (Salkowski’s test)

For the identification of Terpenoids, powdered plant material was dissolved in 5 mL of aqueous solution and boiled in a water bath and then allowed to cool. The solution is filtered, and 2 mL of chloroform was added to the filtrate in a test tube; followed by addition of 3 mL of concentrated sulphuric acid. The appearance of reddish brown interface indicated the presence of terpenoid in the sample.

### High performance liquid chromatography-Diaode Array detection (HPLC-DAD) analysis

#### Samples preparation

The extract sample for HPLC was prepared by mixing 20 mL of hydromethanolic mixture (1:1 *v*/v) with 1 g of ON-Cr. The resultant mixture was heated in a water bath at 70 °C for 1 h with subsequent cooling. Thereafter, the sample was centrifuged for 10 min at 4000 rpm. Finally, 2 mL of test sample was filtered through a Whatman filter paper into HPLC vials.

#### HPLC–DAD procedure

An Agilent 1260 infinity high-performance liquid chromatography (HPLC) system provided with ultraviolet array detector (UVAD), quaternary pump, auto-sampler and degasser was used. Separation was done using an Agilent Zorbax Eclipse XDB-C18 column. A gradient system consisting of solvent B (methanol: acetic acid; deionized water, 100: 20: 180, *v*/v) and solvent C (methanol: acetic acid: deionized water, 900: 20: 80, v/v) was used. The gradient program was started with 100% B at 0 min, 85% B at 5 min, 50% B at 20 min, 30% B at 25 min, and 100% C for 30–40 min [47]. Using this sequence, elution occurred after 25 min. For analysis of compounds, the ultraviolet array detector (UVAD) was set at 280 nm and the spectra were recorded from 190 to 500 nm. Two standard compounds, rutin and pyrogallol were detected in the HPLC analysis.

#### Antioxidant assays

##### 1,1-diphenyl, 2-picrylhydrazyl (DPPH) free radical scavenging assay

ON-Cr was tested for DPPH free radical scavenging assay as previously reported [35, 48]. Briefly, 0.004% DPPH reagent solution was prepared and added to increasing concentration of ON-Cr extract (125, 250, 500 and 1000 μg/mL) with subsequent incubation for 30 min. The absorption of reaction mixture was measured spectrophotometrically (UV-3000 O.R.I. Germany) at 517 nm. Ascorbic acid was used as positive control. The percent scavenging activity of samples was calculated using the following equation:

$$ \% scavenging activity=\frac{\mathrm{absorbance}\  \mathrm{of}\  \mathrm{control}-\mathrm{absorbance}\  \mathrm{of}\  \mathrm{plant}\  \mathrm{extract}}{\mathrm{absorbance}\  \mathrm{of}\  \mathrm{control}}\times 100 $$

The experiment was performed in triplicate. The IC_50_ values were calculated using GraphPad Prism® (version 4.0, Sandiego, CA, USA).

##### 2, 2-Azinobis [3-ethylbenzthiazoline]-6-sulfonic acid (ABTS) free radical scavenging assay

For further evaluation of *Olax nana* antioxidant potentials, ABTS free radical scavenging assay was performed [41, 49]. The principle of this assay is based on the test sample ability to scavenge ABTS free radical cations, ultimately leads to reduced absorbance at 734 nm. After mixing, K_2_S_2_O_8_ (2.45 mM) and ABTS (7 mM), the solution was kept in dark for 12–16 h to form ABTS free radical cations. A 300 μL of ON-Cr test sample (1000 μg/mL-31.25 μg/mL) was gently mixed with ABTS solution (3.0 mL) in a cuvette. The absorbance of the mixture was measured after every 1 min, for a period of 6 min continuously on a UV-VIS spectrophotometer (UV-3000 O.R.I. Germany). As a positive control, ascorbic acid was used. The assay was performed in triplicate and calculation of percent inhibition was done using the following equation:

$$ \% scavenging effect=\frac{control absorbance- sample absorbance}{control absorbance}\times 100 $$

The percent inhibition and EC_50_ value (extract concentration required for 50% reduction of ABTS radicals) were then calculated for expression of antioxidant potential.

##### Hydrogen peroxide (H_2_O_2_) free radicals scavenging assay

As per our previously reported method, the H_2_O_2_scavenging activity of ON-Cr was elucidated [50]. A solution of H_2_O_2_ (2 mM) was prepared in phosphate buffer (50 mM, pH 7.4). In a 0.3 mL of phosphate buffer solution (50 mM), 0.1 mL of test sample was added, which was followed by addition of 0.6 mL of H_2_O_2_ and then vortexed. The absorbance of solution was measured at 230 nm after a period of 10 min (UV-3000 O.R.I. Germany), in comparison to blank. The following equation was used for the calculation of H_2_O_2_ free radical scavenging activity:

$$ \mathrm{Hydrogen}\  \mathrm{peroxide}\  \mathrm{scavenging}\  \mathrm{activity}=\frac{1- sample absorbance}{absorbance of control}\times 100 $$

#### Anticholinesterase assays

The Ellman’s assay was performed to assess the AChE and BChE enzymes inhibition potentials of ON-Cr [51]. The principle of anticholinesterase assay is based on hydrolysis of acetylthiocholine iodide and butyrylthiocholine iodide by respective enzymes, leading to the formation of 5-thio-2-nitrobenzoate anion, which complexes with DTNB to form a yellow color compound, after evaluation by UV-VIS spectrophotometer (UV-3000 O.R.I. Germany).

##### Preparation of solutions

Phosphate buffer solution (0.1 M and pH: 8.0) was prepared as previously reported [48]. For adjusting the pH, potassium hydroxide was used. The final concentrations of 0.03 U/mL and 0.01 U/mL were obtained for AChE (518 U/mg solid) and BChE (7–16 U/mg) after respective dilution in the freshly prepared buffer pH 8.0. Using distilled water, DTNB (0.0002273 M), acetylthiocholine iodide and butyrylthiocholine iodide (0.0005 M) solutions were prepared and stored in a refrigerator (8 °C). The respective dilutions were also prepared for galantamine (positive control) in methanol.

##### Spectroscopic analysis

In a cuvette containing 205 μL of ON-Cr extract, 5 μL of enzyme solution was added, followed by addition of 5 μL DTNB reagent. In a water bath, the resulting solution was incubated for 15 min at 30 °C, with addition of substrate solution (5 μL) subsequently. Furthermore, using a UV-VIS spectrophotometer (UV-3000 O.R.I. Germany) the absorbance was calculated at 412 nm. As a standard inhibitor of cholinesterase, 10 μg/mL of galantamine was used as positive control, while the components other than ON-Cr extract were considered as negative control. At 30 °C for 4 min of reaction time, the absorbance values at specific intervals were noted. The assays were conducted in triplicate, while the change in absorption rate with time gave calculations for the % enzyme activity and % enzyme inhibition by galantamine and ON-Cr sample.

#### α- glucosidase inhibitory assay

The protocol of McCue et al., was adopted for the assessment of ON-Cr α-glucosidase inhibitory potential [52]. An enzyme solution was prepared by mixing 120 μL of phosphate buffer (0.1 M, pH 6.9) with α-glucosidase enzyme (20 μL of 0.5 unit/mL). The solution of substrate having *p*-nitrophenyl-α-D-glucopyranoside was prepared in phosphate buffer (0.1 M, pH 6.9). The ON-Cr test sample was prepared in a concentration range of 31.25–1000 μg/mL followed by mixing with enzyme solution and incubation for 15 min at 37 °C. Furthermore, to the mixture of enzyme and test sample, substrate solution (20 μL) was added and incubated under the same conditions. The reaction was completed by the addition of sodium carbonate solution (80 μl of 0.2 M). Using a UV-VIS spectrophotometer (UV-3000 O.R.I. Germany), the absorbance of samples was recorded at 405 nm. As a positive control, acarbose was used while the sample devoid of α-glucosidase was used as a blank. The experiment was performed in triplicate, while the following equation was used for the calculation of percent inhibition:

$$ \% inhibition=\frac{control absorbance- sample absorbance}{control absorbance}\times 100 $$

The concentration of ON-Cr extract which inhibited substrate hydrolysis via AChE and BChE enzymes by 50% (IC_50_ value) was calculated from the dose response curve [53]. The kinetics of enzyme activity was measured in the presence of increasing concentrations of extract.

### Statistical analysis

All the assays were performed three times, while values were represented as ± S.E.M. One-way ANOVA followed by Bonferroni’s multiple comparison post hoc test was used for the analysis of antioxidant activity and enzyme inhibition assays. At 95% confidence interval, a *P* value of <0.05 was considered as statistically significant. Lineweaver-Burk plots (1/v versus 1/[s]) where v is reaction velocity and [s] is substrate concentration were plotted from assays using a range of plant extract concentrations. The V_max_ and K_m_ values were determined using Michaelis-Menten kinetics.

## Results

### Phytochemical analysis

**Fig. 2:**
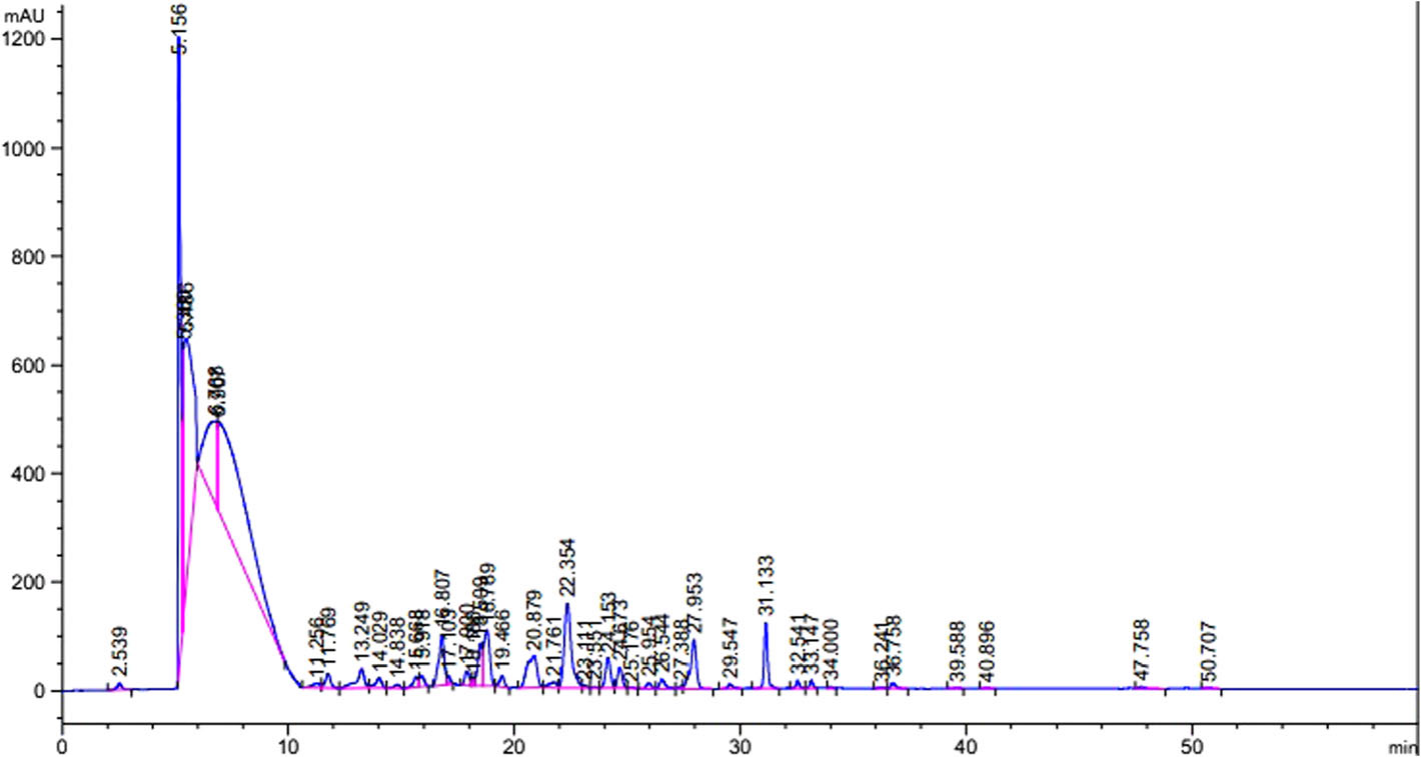
HPLC-DAD chromatogram of methanolic extract of *Olax nana*

The preliminary phytochemical analysis of ON-Cr revealed the presence of various secondary metabolites like alkaloids, flavonoids, tannins, sterols, saponins and terpenoids. Table 1 highlights the observational results of all the phytochemical tests. In the HPLC-DAD analysis of ON-Cr, several components were separated in different ratios but gallic acid derivative (25.57%), hydroxybenzoic acid derivative (17.81) and rutin (5.35%) were compounds present in higher concentrations as summarized in Fig. 2 and Table 2. The highest concentrated signals were observed at retention times of 6.9, 5.8, 5.2, 6.3 min which were 25.57, 17.81, 14.41 and 8.19% respectively.

**Table 1:** Different phytochemical tests for aqueous methanolic extract of *Olax nana*

S. No	Phytochemical tests	Observations	Results
1	Alkaloids	ppt.	+
2	Flavonoids	Formation of yellow color which changed to colorless on acid addition	+
3	Glycosides	Reddish ppt. is not formed	-
4	Tannins	Formation of bluish-black color	+
5	Sterols	green to pink color was absent	+
6	Saponins	frothing bubbles are formed	+
7	Anthraquinones	Red, violet or pink color are not formed in aqueous layer	-
8	Terpenoids	Appearance of reddish brown color	+

**Table 2:** HPLC-DAD identification of phenolic compounds in *Olax nana* leaves

Peak	RT (min)	Peak height (mAU)	Peak area %	Proposed identity of compound*	HPLC–DAD λmax (nm)	References
1	2.5	13.29053	0.320	Ascorbic Acid	244	Mradu *et al.,* 2012
2	5.2	1168.32886	14.41	Gallic acid derivative	273, 279, 288	Aaby *et al.,* 2007
3	5.3	504.33762	3.19	Gallic acid derivative	280	Mradu *et al.,* 2012
4	5.8	456.65558	17.81	Hydroxybenzoic acid derivative	280	Santos *et al.,* 2014
5	6.3	151.39925	8.20	Hydroxybenzoic acid derivative	274	Santos *et al.,* 2014
6	6.9	163.31013	25.57	Gallic acid derivative	271, 278, 287	Aaby *et al.,* 2007
7	11.1	8.41879	0.33	Kaempferol-7-O-glucoside	254	Ibrahim *et al.,* 2015
8	11.6	26.59516	0.70	*p*-Coumaric acid derivative	313	Santos *et al.,* 2014
9	13.2	35.75774	1.36	Isovitexin-4-O-glucoside	254	Ibrahim *et al.,* 2015
10	14.3	18.4162	0.57	Caftaric acid	242; sh 298; 328	Carazzone *et al.,* 2013
11	14.9	6.63179	0.17	Gallic acid derivative	280	Santos *et al.,* 2014
12	15.5	19.11495	0.46	Hydroxybenzoic acid derivative	278	Santos *et al.,* 2014
13	15.7	19.45154	0.43	Hydroxybenzoic acid derivative	278	Santos *et al.,* 2014
14	16.9	92.68282	2.47	p-Hydroxybenzoic acid	256	Santos *et al.,* 2014
15	17.0	15.39486	0.23	Caffeoylmalic acid	327, 300, 268	Santos *et al.,* 2014
16	17.5	26.84283	0.60	bis-HHDP-glucose	232	Aaby *et al.,* 2007
17	18.1	14.13607	0.21	Quercetin-3-O-triglucoside	268; 340	Lin *et al.,* 2011
18	18.3	77.88322	1.70	Galloyl-HHDP-glucose	232	Aaby *et al.,* 2007
19	18.9	102.11224	2.93	Apigenin-7-O-rutinoside	254	Ibrahim *et al.,* 2015
20	19.9	19.88177	0.46	P-coumaric acid derivative	228, 316	Santos *et al.,* 2014
21	20.7	57.97879	2.78	Vanillic acid	260; 292	Santos *et al.,* 2014
22	21.4	10.30173	0.39	Caffeic acid	238; 298 sh; 323	Santos *et al.,* 2014
23	22.3	89.48333	5.35	Rutin	155.3661	Reference Standard
24	23.2	4.74129	0.10	Syringic acid	274	Santos *et al.,* 2014
25	23.5	2.06536	0.04	Proanthocyanidin trimer	284	Aaby *et al.,* 2007
26	24.1	54.95284	1.32	Quercetin-di-glucoside	256; sh 268; 350	Llorach *et al.,* 2008
27	24.7	38.34863	0.87	Quercetine glycoside	256; sh 266; 354	Llorach *et al.,* 2008
28	25.4	1.90931	0.04	Quercetine glycoside	256; sh 266; 354	Llorach *et al.,* 2008
29	25.9	11.029	0.26	Caffeoylmalic acid	244; sh 298; 328	Llorach *et al.,* 2008
30	26.6	17.19059	0.49	p-Coumaric acid	228; 310	Santos *et al.,* 2014
31	27.953	5.04913	0.09	Pyrogallol	244; sh 298; 328	Reference Standard
32	27.7	89.48333	2.58	Chicoric acid	244; sh 298; 328	Carazzone *et al.,* 2013
33	29.2	8.83803	0.22	Caffeic acid derivative	242; sh 298; 322	Santos *et al.,* 2014
34	31.3	120.93526	2.08	Quercetin-3-(caffeoyldiglucoside)- 7-glucoside	252; sh 268; 332	Santos *et al.,* 2014
35	32.7	14.63641	0.26	Quercetin-3-d-galactoside	256; 268 sh; 356	Santos *et al.,* 2014
36	33	14.05107	0.24	Quercetin-3-O-glucoside	256; 268 sh; 356	Santos *et al.,* 2014
37	34	3.52791	0.06	Rosmarinic acid	330; 290 sh	Santos *et al.,* 2014
38	36.3	2.60122	0.08	Quercetin-3-feruloylsophoroside	256; sh 268; 334	Lin *et al.,* 2011
39	39.6	2.02323	0.06	Rosmarinic acid derivative	290; 328	Santos *et al.,* 2014
40	40.5	2.04721	0.04	cyanidin-3-glucoside	278sh; 232	Aaby *et al.,* 2007

*The compounds were identified by comparing absorption spectra of the sample with the standard compounds or from the values reported in the literature

### Antioxidant assays

**Fig. 3:**
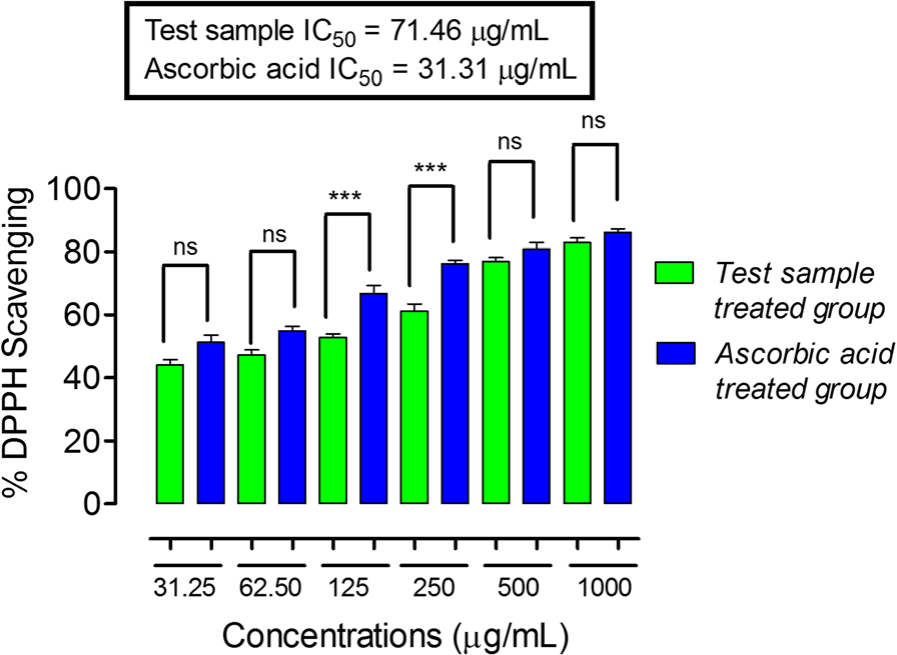
Results of antioxidant potentials of *Olax nana* using DPPH assay. Values significantly different in comparison to the positive control (ascorbic acid) and test samples at the same concentration at 95% confidence interval. ^***^ *P* < 0.001, ns: values not significantly different in comparison to % inhibition of the positive control (*P* > 0.05) at the same concentration. One-way ANOVA followed by Bonferroni’s multiple comparison post hoc test

**Fig. 4:**
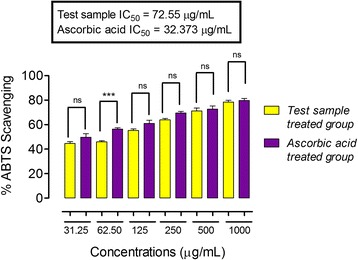
Results of antioxidant potentials of *Olax nana* using ABTS free radicals scavenging assay. Values significantly different in comparison to the positive control (ascorbic acid) and test samples at the same concentration, at 95% confidence interval. ^***^ *P* < 0.001, ns: values not significantly different in comparison to % inhibition of the positive control (*P* > 0.05) at the same concentration. One-way ANOVA followed by Bonferroni’s multiple comparison post hoc test

**Fig. 5:**
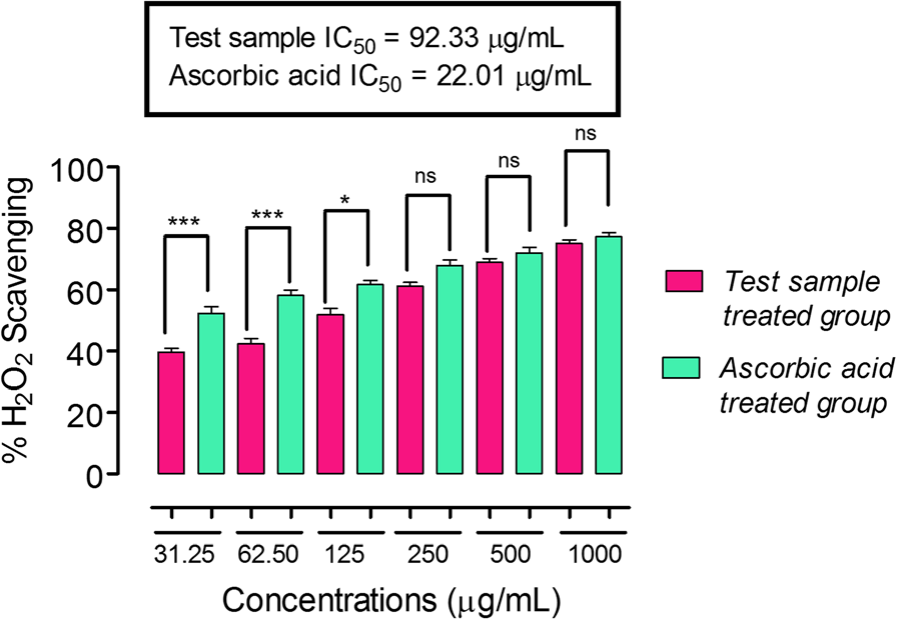
Results of antioxidant potentials of *Olax nana* using H_2_O_2_ anti-radicals assay. Values significantly different in comparison to the positive control at the same concentration i.e. ^***^ *P* < 0.001, ^**^ *P* < 0.01, ns: values not significantly different in comparison to % inhibition of the positive control (*P* > 0.05) at the same concentration. One-way ANOVA followed by Bonferroni’s multiple comparison post hoc test

In the DPPH free radical scavenging assay, ON-Cr at the concentrations of 31.25, 62.50, 500 and 1000 μg/mL demonstrated % inhibition not significantly different when compared to the positive control (ascorbic acid) (*P* > 0.05) at the same concentration. As shown in Fig. 3, the IC_50_ value of ON-Cr was 71.46 μg/mL, while that of ascorbic acid was 31.31 μg/mL. Interestingly, in ABTS free radicals scavenging assay, ON-Cr only at the concentration of 62.05 μg/mL demonstrated significantly different % inhibition in comparison to that of ascorbic acid (*P* < 0.001 at 95% confidence interval). While at the concentrations of 31.25, 125, 250, 500 and 1000 μg/mL, the % inhibition values were not significantly different in comparison to ascorbic acid at similar concentrations (*P* > 0.05 at 95% confidence interval). As shown in Fig. 4, the IC_50_ value of ON-Cr was 72.55 μg/mL, while that of ascorbic acid (positive control) was 32.37 μg/mL. In the H_2_O_2_ anti-radicals assay, ON-Cr at the concentrations of 31.25, 62.50 μg/mL demonstrated % inhibition significantly different in comparison to that of ascorbic acid (*P* < 0.001 at 95% confidence interval). While at the concentrations of 125, 250, 500 and 1000 μg/mL, the % inhibition values were not significantly different when compared to ascorbic acid at similar concentrations (*P* > 0.05 at 95% confidence interval). As shown in Fig. 5, the IC_50_ value of ON-Cr was 92.33 μg/mL, while that of ascorbic acid was 22.01 μg/mL.

### Anticholinesterase assays

**Fig. 6:**
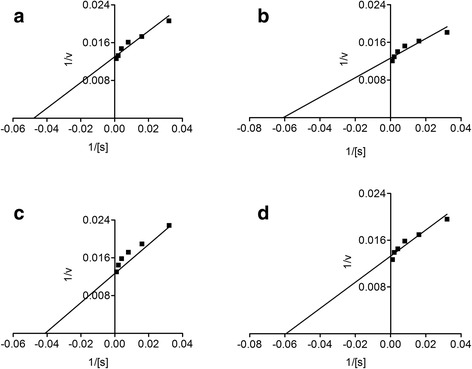
Lineweaver-Burk plots representing the reciprocal of initial enzyme velocity versus the reciprocal of substrate concentration in the presence of different concentrations of ON-Cr and the positive control galantamine for acetylcholinesterase (**a** and **b**) and butyrylcholinesterase (**c** and **d**), respectively

**Fig. 7:**
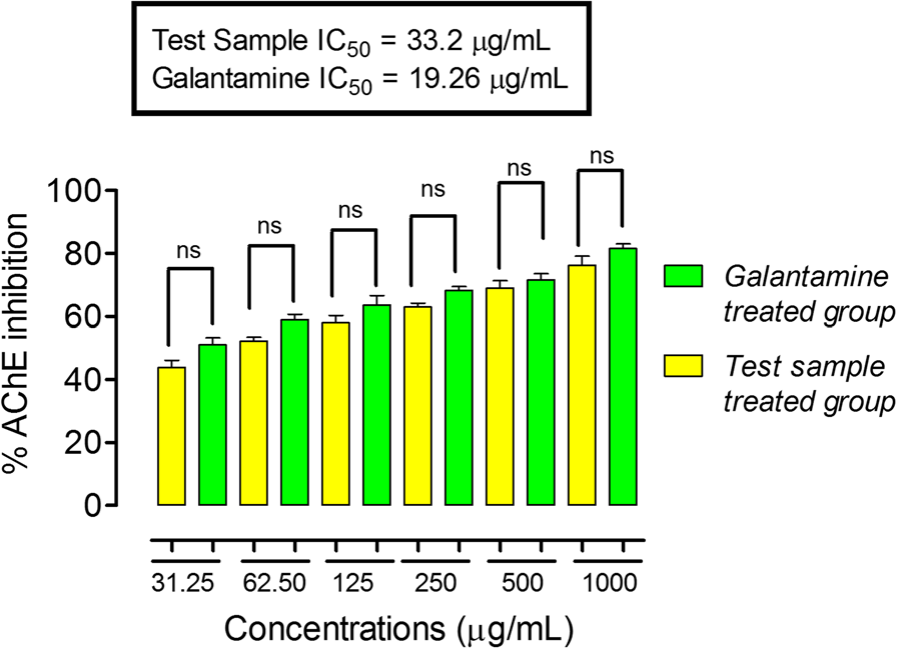
Acetylcholinesterase inhibitory activity (AChE) of aqueous methanolic extract of *Olax nana* using Ellmen's assay. One-way ANOVA followed by Bonferroni’s multiple comparison post hoc test was used for statistical significant different between the standard drug (galantamine) and test sample at the same concentration, at 95% confidence interval. ns: values not significantly different in comparison to % inhibition of the standard drug (*P* >0.05) at the same concentration

**Fig. 8:**
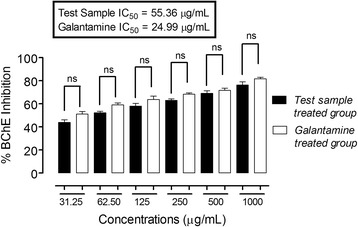
Butyrylcholinesterase inhibitory activity (BChE) of aqueous methanolic extract of Olax nana using Ellmen's assay. One-way ANOVA followed by Bonferroni’s multiple comparison post hoc test was used for statistical significant different between the standard drug (galantamine) and test sample at the same concentration, at 95% confidence interval. ns: values not significantly different in comparison to % inhibition of the standard drug (*P* >0.05) at the same concentration

In the acetylcholinesterase inhibitory activity, the IC_50_ value for ON-Cr was 33.2 μg/mL, while that of galantamine was 19.26 μg/ mL (Fig. 6). Furthermore, in butyrylcholinesterase inhibitory activity, ON-Cr demonstrated potential IC_50_ value of 55.36 μg/mL, while that of galantamine was 24.99 μg/mL (Fig. 7). The strong inhibitory propensity of ON-Cr was further corroborated from the Linewear-Burk plots (Fig. 8) in which ON-Cr prospectively inhibited the enzymatic activities of both AChE and BChE, the inhibitory effect was comparable to that of the positive control, galantamine. The K_m_ value of the substrates, ON-Cr and galantamine for AChE were 20.94 and 16.43 μg while the V_max_ values were 77 and 79.03 μg/min, respectively. Similarly, for BChE, the K_m_ values of ON-Cr and galantamine were noted as 24.32 and 16.86 μg, while the V_max_ values were calculated as 73.22 and 75.35 μg/min, respectively.

### α- glucosidase inhibitory assay

**Fig. 9:**
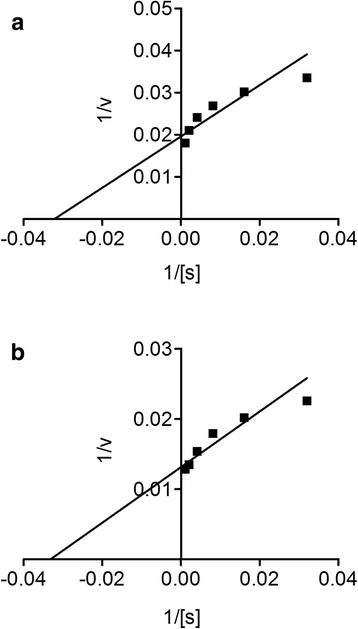
Lineweaver-Burk plots representing the reciprocal of initial α-glucosidase velocity versus the reciprocal of substrate concentration in the presence of different concentrations of ON-Cr (**a**) and the positive control, galantamine (**b**)

The percent inhibition of α-glucosidase via ON-Cr was found to be dose-dependent. The IC_50_ value of ON-Cr was 639.89 μg/mL, while that of acarbose used as positive control was 61.19 μg/mL. The percent inhibition of α-glucosidase of ON-Cr in a concentration range of 31.25–1000 μg/mL is shown in Table 3. The Linewear-Burk plots for the inhibition of α-glucosidase by ON-Cr and galantamine is shown in Fig. 9. The dissociation constant (K_m_) and V_max_ values of ON-Cr for α-glucosidase were 31.11 μg and 51.01 μg/min, respectively, which were comparable to that of positive control, acarbose (30.19 μg and 76.17 μg/min), thus showing potent inhibitory proclivity against α-glucosidase.

**Table 3:** α-Glucosidase inhibitory activity of *Olax nana*

Concentration (μg/mL)	% inhibition of extract	IC_50_ (μg/mL)	Acarbose (μg/mL)	IC_50_ (μg/mL)
1000	55.24±0.33	639.89	77.76±0.17	61.19
500	47.35±0.49		73.99±0.08	
250	41.45±0.23		64.97±0.17	
125	37.18±0.09		55.65±0.43	
62.5	33.08±0.22		49.50±0.45	
31.25	29.79±1.12		44.21±0.26

## Discussion

In the present study, detailed phytochemical investigation and pharmacogonostic activity of *Olax nana* aqueous methanolic extract has been reported for the first time. Presence of various secondary metabolites like alkaloids, flavonoids, tannins, sterols, saponins and terpenoids have been found in the preliminary phytochemical analysis. Interestingly, in the HPLC-DAD results the highest concentrated signals were of gallic acid derivatives, hydroxybenzoic acid derivatives and rutin. Previously, *Olax scandens* has been reported for the presence of various essential phytochemicals like oleanolic acid, octacosanol, aleanolic acid and β-sitosterol etc. [54]. Furthermore, chemical investigation of *Olax mannii* Oliv also reported to contain useful flavonoid glycoside and derivatives [30].

Free radicals are generated during metabolic processes in the body and are implicated in a variety of disorders including neurodegenerative disorders, coronary heart disease, cancer, diabetes and immune-suppression [55, 56]. The hydrogen peroxide and singlet oxygen are included in the list of non-free radicals, while nitric oxide, lipid peroxyl, superoxide and hydroxyl are common free radicals [57]. Naturally, the lethal effect of free radicals is nullified in our body via various defense mechanisms, including the protective antioxidant system and chain breaking antioxidants generation [58]. Furthermore, extensive tissue injury occurs when free radical generation rate surpasses the limit of natural scavenging mechanisms. Hence, many diseases including neurogenerative disorders can be cured with the help of drugs having free radical scavenging ability. Plants are a valuable resource of antioxidants which have been reported to protect from free radicals induced damage [59]. Significant anti-radical activities was revealed by ON-Cr in the DPPH, ABTS and H_2_O_2_ free radical scavenging assays with IC50 values of 71.46, 72.55 and 92.33 μg/mL, respectively.

In the treatment of neurological disorders, potential inhibitors of acetylcholinesterase (AChE) and butyrylesterase (BChE) enzymes are of prime importance. These enzymes are involved in the catabolism of acetylcholine (ACh), hence have applications in the treatment of Alzheimer’s disease (AD) [60], along with many other neurological disorders [61]. AD is connected with memory impairment, change of behavior and cognitive dysfunction [62]. A decline in the amount of ACh via malfunctioning of various biochemical pathways ultimately leads to AD [63]. AChE along with BChE result in the termination of signal transmission carried out in the synapse via ACh. Hence, by inhibiting these key metabolizing enzymes, we can treat AD and many other neurological disorders [64]. Unfortunately, the available drugs are associated with hepatotoxicity along with many adverse side effects and show effectiveness only in mild type of AD [65]. Hence, it’s of prime importance to explore novel remedies for AD, which is effective and have less side effects propensities. Plants are widely explored for potential therapeutic compounds effective in the treatment of neurological disorders [66]. Many research groups including ours have also explored the potential of medicinal plants as valuable source for the treatment of neurodegenerative disorders especially AD [35, 67]. In the current study, very potent acetylcholinesterase (AChE) and butyrylesterase (BChE) inhibition potential was demonstrated by ON-Cr. Interestingly, in both acetylcholinesterase and butyrylcholinesterase inhibitory activities of ON-Cr, all the concentrations (31.25, 62.50, 125, 250, 500 and 1000 μg/mL) demonstrated % inhibition values not significantly different in comparison to galantamine (standard drug) at the same concentration (*P* > 0.05 at 95% confidence interval). Previously, *Olax Subscorpioidea* is also reported to possess neuroprotective, anxiolytic and anti-depressant properties [18, 21]. Significant results noticed in the antioxidant and anticholinesterase assays of ON-Cr may be attributed to the predominant presence of Gallic acid derivatives among the identified compounds (Table 2), which is a common poly phenol and strong antioxidant having promising neuroprotective potentials [68, 69]. The plant extracts also contain ascorbic acid which is a strong antioxidant. Quercetin and its derivatives are extensively studied as neuroprotective and antioxidant agents [70–72]. ON-Cr contains several quercetin derivatives which may be responsible for the antioxidant and cholinesterase inhibitory potentials of extracts samples. Pyrogallol, another polyphenolic compound was identified via comparison with the standards. This compound is a potent auto-oxidant and stimulates the activation of indigenous antioxidant system [73]. Rutin has a well established antioxidant and neuroprotective potentials [74, 75]. The current antioxidant and anti-cholinesterase potentials of ON-Cr can be attributed to the presence of these compounds. Yet further studies regarding the isolation and testing of the plant samples will validate its potential use as a neuroprotective agent in folk medicine.

In the early stage of diabetes mellitus type 2, postprandial hyperglycemia occurs due to impaired secretion of insulin after meal. It is believed that free radicals may cause hyperglycemia and may result in complications, like retinopathy, nephropathy, and memory impairment [76]. The dietary carbohydrates in our meal are digested by a group of enzymes known as glucosidases. Acarbose is one of the key inhibitor of glucosidase, which ultimately downregulate the process of carbohydrate digestion and delays its absorption into the blood. Hence, potential inhibitors of glucosidases can ultimately prevent type 2 diabetes mellitus development via lowering of glucose after meal [77]. Plants have been reported as a great source of these type of inhibitors [78, 79]. Our results showed interesting inhibition of α-glucosidase via ON-Cr. The dissociation constant (Km) and Vmax values as reported by ON-Cr and acarbose were comparable to each other (Fig. 9). To the best of our knowledge, this is the first report of α-glucosidase inhibition via any Olax specie.

## Conclusions

This present research is an exclusive report on the phytochemical composition, antioxidant, cholinesterase and α-glucosidase inhibitory potentials of *Olax nana* methanolic extract. The results of enzyme inhibitions and anti-radical activities were highly significant. These results indicate that *Olax nana*, is highly enriched with potential antioxidant compounds which can be exploited as potential therapy for the treatment of many disorders. In light of the potential biomedical application as demonstrated by the above results, further studies on the bioassay-guided isolation of potential active compounds and their therapeutic evaluation is recommended.
